# Experimental investigation of the effective point of measurement for plane‐parallel chambers used in electron beam dosimetry

**DOI:** 10.1002/acm2.14059

**Published:** 2023-06-12

**Authors:** Kohki Yasui, Yujiro Nakajima, Yuhi Suda, Yu Arai, Takuto Takizawa, Kaito Sakai, Yukio Fujita

**Affiliations:** ^1^ Department of Radiological Sciences Komazawa University Graduate School Setagaya‐ku Tokyo Japan; ^2^ Department of Radiation Oncology Tokyo Metropolitan Cancer and Infectious Diseases Center Komagome Hospital Bunkyo‐ku Tokyo Japan; ^3^ Department of Radiological Sciences Komazawa University Setagaya‐ku Tokyo Japan

**Keywords:** effective point of measurement, electron beam dosimetry, ion chamber, microDiamond

## Abstract

In this study, the effective point of measurement (EPOM) for plane‐parallel ionization chambers in clinical high‐energy electron beams was determined experimentally. Previous studies have reported that the EPOM of plane‐parallel chambers is shifted several tens of millimeters downstream from the inner surface of the entrance window to the cavity. These findings were based on the Monte Carlo (MC) simulation, and few experimental studies have been performed. Thus, additional experimental validations of the reported EPOMs were required. In this study, we investigated the EPOMs of three plane‐parallel chambers (NACP‐02, Roos and Advanced Markus) for clinical electron beams. The EPOMs were determined by comparing the measured percentage depth‐dose (PDD) of the plane‐parallel chambers and the PDD obtained using the microDiamond detector. The optimal shift to the EPOM was energy‐dependent. The determined EPOM showed no chamber‐to‐chamber variation, thereby allowing the use of a single value. The mean optimal shifts were 0.104 ± 0.011, 0.040 ± 0.012, and 0.012 ± 0.009 cm for NACP‐02, Roos, and Advanced Markus, respectively. These values are valid in the *R*_50_ range from 2.40 to 8.82 cm, which correspond to 6–22 MeV. Roos and Advanced Markus exhibited similar results to those of the previous studies, but NACP‐02 showed a larger shift. This is probably due to the uncertainty of the entrance window of NACP‐02. Therefore, it is necessary to carefully consider where the optimal EPOM is located when using this chamber.

## 1 INTRODUCTION

Current dosimetry protocols^1^, ^2^ for clinical high‐energy electron beams necessitate the use of plane‐parallel chambers instead of cylindrical chambers, particularly at low energies. The measurement point of the plane‐parallel chamber is the inner surface of the entrance window, and the shape of the electron fluence spectrum at this position is considerably similar to that in the phantom at the measurement point. Therefore, the replacement correction factor *p*_repl_ was considered to be unity for a plane‐parallel chamber with a sufficiently large guard ring. Based on this, the effective point of measurement (EPOM) of the chamber is on the inner surface of the entrance window, at the center of the window, and no correction using the perturbation factors (*p*_Q_) is required.

According to recent studies, the EPOMs of plane‐parallel chambers are shifted several tens of millimeters downstream from the inner surface of the entrance window to the cavity.^3^, ^4^, ^5^, ^6^, ^7^ This is because *p*_repl_, which was assumed to be unity, and *p*_wall_, which explains the influence of the chamber wall, vary considerably with the measurement depth,^8^, ^9^ indicating that the EPOM minimizing the depth variation with respect to the perturbation factor is not present on the inner surface of the entrance window. Studies^10^, ^11^ proposing novel calibration methods should employ optimal EPOMs. To address the aforementioned issues, optimal EPOM positions must be determined.

Majority of previous studies on the EPOM were based on the Monte Carlo (MC) simulation^4^, ^5^, ^6^, ^7^; however, experimental data on the EPOM for several plane‐parallel chamber types are considerably limited. The MC method has been extensively employed to precisely compute dosimetric quantities. However, numerous sources of uncertainty, such as geometrical variations, are associated with MC simulation. There are substantial variations in the manufacturing of plane‐parallel chambers.^12^ The MC simulation of the ionization chamber model considers an idealized geometry with no variability in the ionization chamber manufacturing process. Geometrical variations can cause a systematic uncertainty. The optimal EPOM estimates based on MC simulations should be validated by comparing them with the experimentally determined results. Some reports show a shift of approximately 0.04 cm toward the cavity from the inner surface of the entrance window in the experimental determination of the EPOM for Roos.^3^, ^13^ In case of NACP‐02, this shift is approximately 0.07 cm; however, this observation has been reported by only one study.^13^ Advanced Markus has not been experimentally investigated to determine the EPOM. Therefore, there are few previous studies, particularly on NACP‐02 and Advanced Markus, and experimentally determined EPOM data are lacking. Further, the chamber‐to‐chamber variation with respect to the EPOM has not been reported by previous studies on experimental determination of the EPOM.

In this study, the EPOM of a plane‐parallel ionization chamber for clinical high‐energy electron beam dosimetry was experimentally determined. We also evaluated the chamber‐to‐chamber variation in the EPOM and determined whether generic chamber type–specific values existed. Further, a previous study used a plastic scintillation detector to acquire the reference data for determining the EPOM of the chamber,^13^ but this detector required correction for Cherenkov light. We discovered that the microDiamond is the most appropriate detector for EPOM measurement.

## 2 METHODS

In Section 2A, we present a detailed summary of the experimental determination of the EPOM. A detector with a mean‐restricted mass collision stopping power ratio that does not change with energy must be used to determine EPOMs, and the selection of this reference detector substantially affects the results. Thus, the measured percentage depth‐dose (PDD) curves of the microDiamond detector were validated via MC simulations. Section 2B describes the details of MC simulations. Three different types of plane‐parallel chambers were examined. Section 2C presents the experimental setup and procedure for measuring PDD curves.

### 2.1 Experimental determination of the effective point of measurement

The EPOMs were determined by comparing the reference PDD curves. This approach is the same as that adopted in previous studies by Wang and Rogers^4^ or Philip von Voigts‐Rhetz et al.^5^ This approach defined the EPOM as a shift Δz from the inner surface of the entrance window that minimizes the root‐mean‐square (RMS) deviation, which is defined as follows:

(1) $$\begin{array}{c} \;{\left(rms\left(\Delta z\right)\right)}^{2}=\frac{1}{n}\;\sum_{i}{\left(D_{w}^{i}\left(z+\Delta z\right)-s_{w,a}^{\Delta }\left(z\right)\cdot {\bar{D}}_{det}\left(z\right)\right)}^{2}, \end{array}$$

where *z* represents the depth, *n* represents the number of measurement points in the evaluation range, $D_{w}^{i}$ represents the absorbed dose to water, $s_{w,a}^{\Delta }$ denotes the water/air mean‐restricted mass collision stopping power ratio with cutoff energy Δ, and ${\bar{D}}_{det}$ denotes the absorbed dose to air obtained from the chamber. Here, the PDD of the microDiamond detector corresponds to $D_{w}^{i}$, whereas the PDD obtained from the chamber corresponds to $s_{w,a}^{\Delta }\cdot {\bar{D}}_{det}$. The minimum ${\left(rms(\Delta z)\right)}^{2}$ was determined by shifting Δz in 0.01 cm intervals. The EPOM was determined using a PDD ranging from 0.1 cm on the phantom surface to $z/R_{50}=\;1.2$. This depth is near the electron practical range, and the bremsstrahlung region deeper than this is not a main electron beam component. Further, Burns' approximation^14^ used to correct for $s_{w,a}^{\Delta }$ is effective in the range of $0.02\leq z/R_{50}\leq 1.2$, which is another reason for setting the evaluation range as aforementioned. This range is reasonable because it is close to the practical range set by Philip von Voigts‐Rhetz et al.^5^ in a previous study.

### 2.2 Monte Carlo simulation of microDiamond detector

To confirm that the response is independent on energy, the PDD of the microDiamond detector was determined via MC simulation. The microDiamond detector was reproduced using the egs_chamber^15^ user code for EGSnrc^16^ based on the specification of the microDiamond detector provided by PTW. All the simulations employed TrueBeam (Varian Medical Systems, Palo Alto, CA, USA) electron beam phase space files. The dose comparison was only performed with an electron beam of 22 MeV energy, which is the maximum electron beam energy of clinically available TrueBeam, because the mean energy of electron beams decreases when traversing a medium. The number of primary electron histories in the simulation was approximately 10^11^ because the sensitive volume of the microDiamond detector was considerably small and several particles were required. At a source‐to‐surface distance (SSD) of 100 cm, the geometry was a 40 × 40 × 30 cm^3^ water phantom and the field size was 20 × 20 cm^2^. The particle production threshold and transport cutoff energy were set to AE = ECUT = 521 keV and AP = PCUT = 10 keV for electrons and photons, respectively. The calculation times were optimized by implementing range‐rejection‐based Russian roulette, which is a variance‐reduction method. The PDD for absorbed dose to water was computed within a small water voxel with a radius of 0.5 cm and a thickness of 0.02 cm in the beam incidence direction. This water voxel had a size similar to that in the previous study,^4^, ^5^ which evaluated the EPOM of the chambers based on MC simulations. The sensitive volume of the microDiamond detector and the water voxels were used to calculate the energy deposition. The dose difference between both results was evaluated in the range of the EPOM determination (from 0.1 cm on the phantom surface to $z/R_{50}$ = 1.2); this covered a wide range of electron energies. Note that no smoothing or filtering was performed on the simulation results because the statistical uncertainties of the microDiamond detector and water results in the range were sufficiently small (<0.5%).

### 2.3 Ionization chambers, experimental setup, and measurement of PDD

Three plane‐parallel ionization chambers, NACP‐02 (SN 9701, 20555, 20557, IBA Dosimetry, Schwarzenbruck, Germany), PTW 34001 Roos (SN 994, 2915, 2956, PTW‐Freiburg, Freiburg, Germany), and PTW 30045 Advanced Markus (SN 413, 1878, 1909, PTW‐Freiburg, Freiburg, Germany), were used for measuring PDD. To examine the chamber‐to‐chamber variations in the EPOM, three chambers with different serial numbers were prepared for each type. Table 1 lists the geometrical information concerning the air cavities for the plane‐parallel chambers. We selected the PTW60019 microDiamond detector (SN 123214, PTW‐Freiburg, Freiburg, Germany) for reference data acquisition.

**TABLE 1:** Nominal dimensions and physical characteristics of the plane‐parallel chambers.

Chamber	Sensitive volume (cm^3^)	Radius (cm)	Entrance window (cm)	Water‐equivalent scaling (g/cm^2^)	Guard ring (cm)
NACP‐02	0.160	0.500	0.060	0.104	0.300
PTW 34001 (Roos)	0.350	0.800	0.112	0.118	0.400
PTW34045 (Advanced Markus)	0.020	0.250	0.130	0.106	0.200

All the PDD curve measurements were conducted in water using a Blue Phantom 2 (IBA Dosimetry, Schwarzenbruck, Germany). A step‐by‐step collection method was used; the step‐size was 0.1 cm; and the sampling time at each measurement point was 3 s. This step‐size was the minimum value configurable in the software. TrueBeam with 6, 12, 18, and 22 MeV electron beams was used in the experiment. The measurement geometry was 100 cm SSD, and the field size was 20 × 20 cm^2^. To enhance the reproducibility of the measurement results, each detector was used for the measurement multiple times for several days. The average PDD curves with multiple measurements were used to determine Δz.

The ion recombination and polarity effect ($k_{S}$, $k_{pol}$) associated with chamber measurements are dependent on depth.^17^, ^18^ Therefore, for each depth, the measured percentage depth ionization was corrected for $k_{S}$ and $k_{pol}$. Both correction factors were calculated according to TRS‐398^2^. $k_{S}$ was determined by the two‐voltage method. We conducted preirradiation using a dose of approximately 5 Gy to reduce the effect of impurities in microDiamond dosimetry.^19^ The microDiamond detector was positioned in the vertical direction, and the measurement point was set to coincide with the sensitive volume located 0.1 cm below the detector tip.^20^

## 3 RESULTS

### 3.1 Energy dependence of microDiamond detector response in the clinical electron beam

**FIGURE 1:**
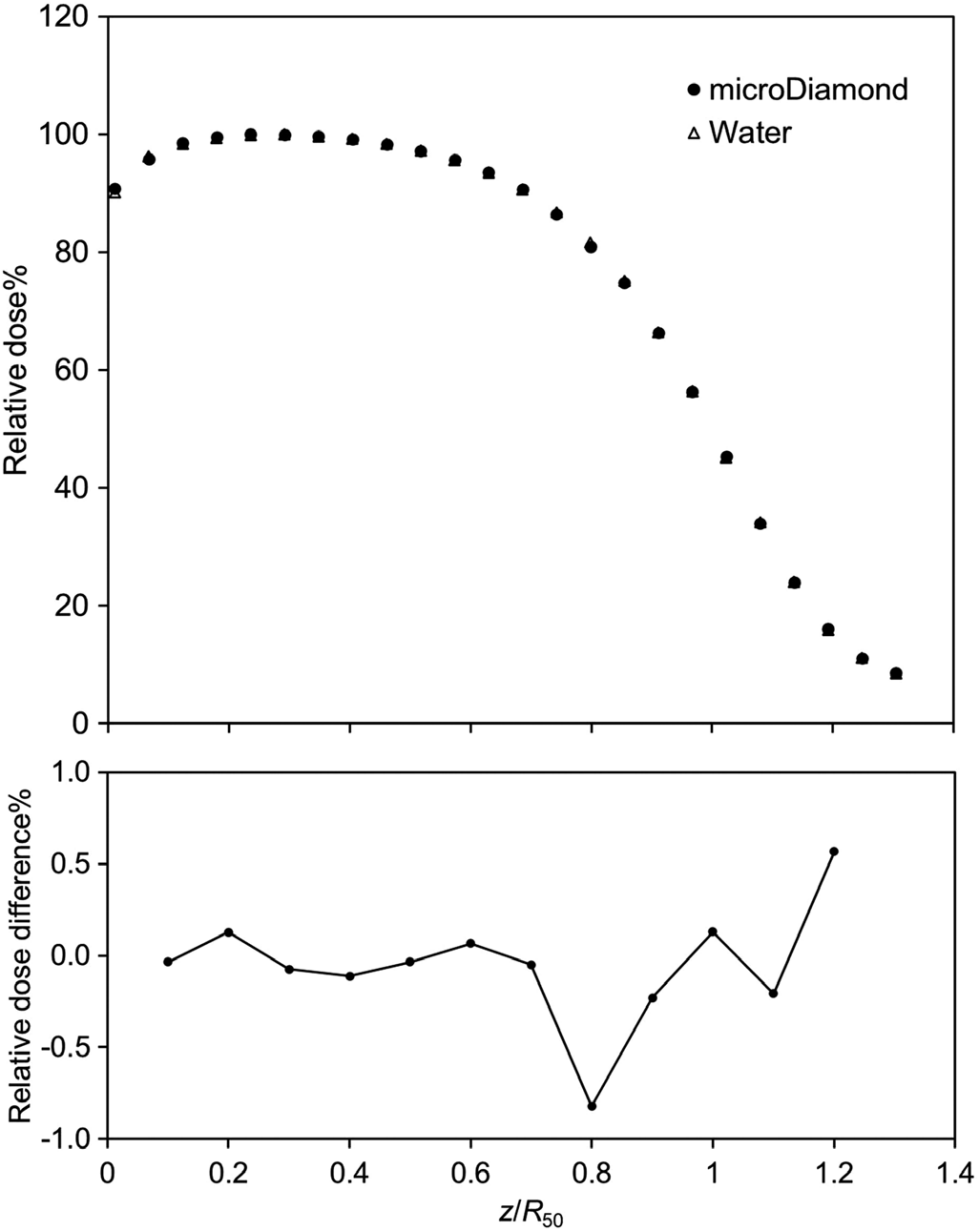
Percentage depth‐dose (PDD) curves (top) and relative dose difference (bottom) at 22 MeV calculated using egs_chamber. The statistical uncertainty is smaller than the plot sizes shown in the figures.

In clinical electron beams, the microDiamond detector showed no energy dependence. Figure 1 shows the calculated PDD curves of the dose to water and the dose to the microDiamond detector in the 22 MeV electron beam. The PDD curves agree within 1% in the evaluation range of the RMS described by Equation (1). These results reveal that no corrections were required for the PDD curves measured using the microDiamond detector, and the PDD curves could be used to calculate the EPOM.

### 3.2 Energy dependence of the effective point of measurement

**FIGURE 2:**
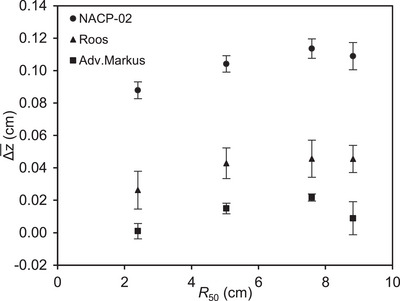
Optimal shift $\bar{\Delta z}$ from the inner surface of the entrance window to the effective point of measurement (EPOM) plotted as a function of *R*_50_. This plot is the mean value of three serial numbers. The error bars show a standard deviation (1σ) resulting from multiple measurements.

The optimal shift exhibited energy dependence. Figure 2 shows the optimal shifts for several beams in case of three chambers as a function of *R*_50_, with a positive shift indicating a shift in the EPOM from the inner surface of the entrance window toward the cavity. Table S1 summarizes the optimal shifts at each energy. Most of the optimal EPOMs were located inside the air cavity, which is a different position from the water‐equivalent thickness. The optimal shift for all chamber types was energy‐dependent, but the highest energy did not exhibit the largest shift. All chamber types had a minimum $\bar{\Delta z}$ at 6 MeV and a maximum at 18 MeV, with a difference of 0.026, 0.020, and 0.021 cm for NACP‐02, Roos, and Advanced Markus, respectively.

### 3.3 Chamber‐to‐chamber variations in the effective point of measurement

**FIGURE 3:**
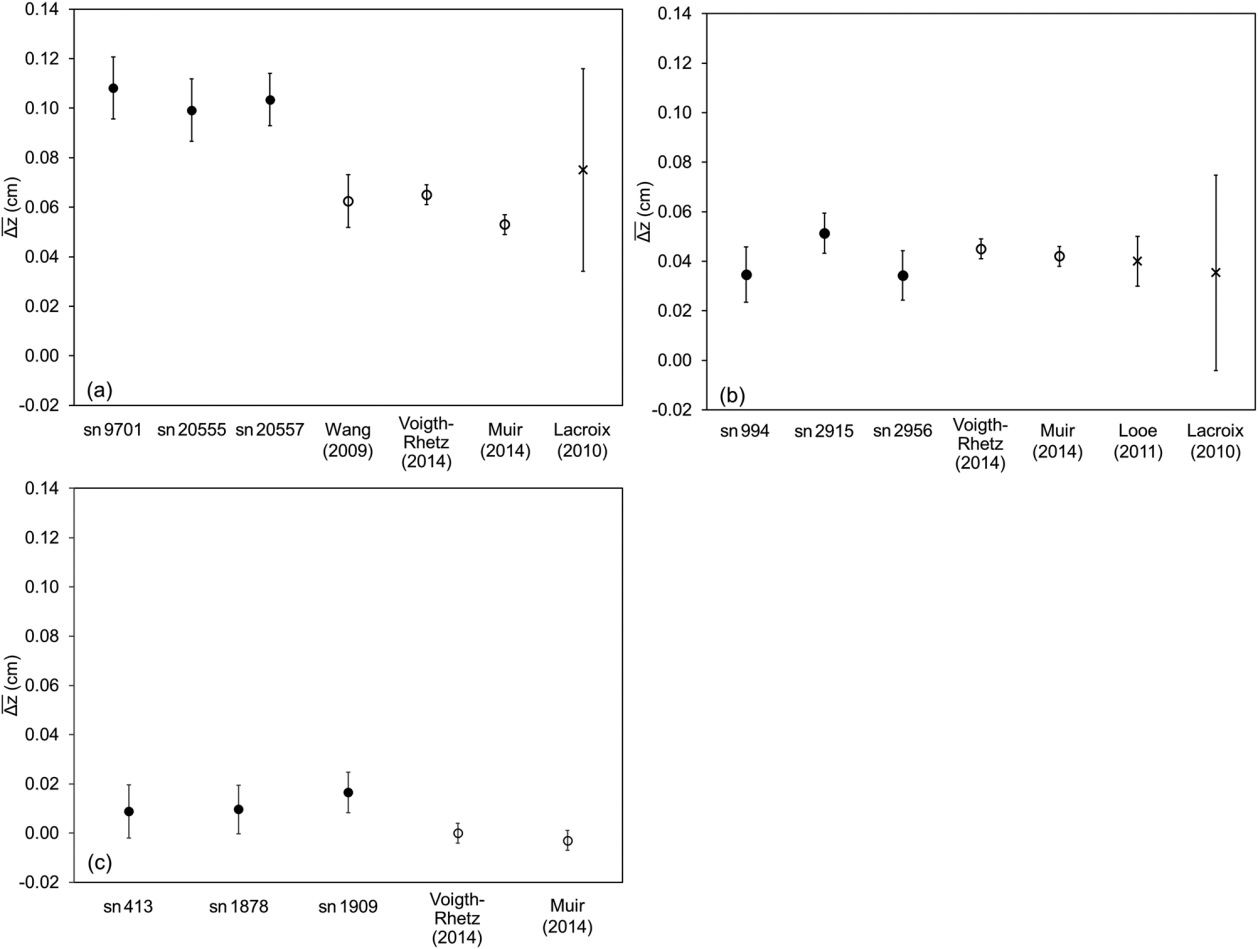
Comparison of the $\bar{\Delta z}$ for different serial numbers: (a) NACP‐02; (b) Roos; (c) Advanced Markus. This finding is the mean of all energies for each serial number. The values reported in previous studies are shown as calculated via the Monte Carlo (MC) simulation (circles) and experimentally determined (crosses). The error bars in this study show 1σ, whereas previous studies based on MC simulation show statistical uncertainties. Wang and Muir's findings for NACP‐02 are plotted based on the specifications.

The optimal shifts exhibited no chamber‐to‐chamber variations. Figure 3 shows the $\bar{\Delta z}$ of different serial numbers and the results of previous studies. The mean shifts in the three serial numbers were 0.104 ± 0.011, 0.040 ± 0.012, and 0.012 ± 0.009 cm for NACP‐02, Roos, and Advanced Markus, respectively. Roos showed a slightly larger chamber‐to‐chamber variation than the other two chamber types. Additionally, the $\bar{\Delta z}$ values for Roos and Advanced Markus agreed with those reported in the previous studies, whereas the $\bar{\Delta z}$ value for NACP‐02 was larger than those previously reported.

## 4 DISCUSSION

The EPOMs of three plane‐parallel chambers were experimentally determined using the measured data of the microDiamond detector. We confirmed that the microDiamond detector is not energy‐dependent and is suitable for reference data acquisition. The optimal shifts showed energy dependency for all chamber types. The chamber‐to‐chamber variations in the EPOM with various serial numbers were consistent within the uncertainties with the mean shifts of the corresponding chamber types.

The microDiamond detector can easily measure PDD curves as reference data without correcting the stopping power ratio. If the microDiamond detector had energy dependence, we would expect a change in the dose difference compared to the water PDD curve. Consequently, the microDiamond detector showed no change in the relative dose difference, even at deeper depths. Pimpinella and Stravato^21^ determined the microDiamond detector response variation in a clinical electron beam via MC calculations. They found that the response variation was within 2% from 6 to 18 MeV and no correction factor was required for relative dosimetry. In this study, only 22 MeV was targeted, but the response variation was within 1%, which agreed with the results reported by Pimpinella and Stravato.^21^ Further, the microDiamond detector has a high resolution with a sensitive volume thickness of 1 μm. A thin sensitive volume thickness can reduce the volume averaging effect in dosimetry with steep dose gradients, such as in electron beam dosimetry. Thus, the microDiamond detector is suitable for reference data acquisition to experimentally determine the EPOM.

The EPOM of the plane‐parallel chamber is located in the air cavity and is energy‐dependent. Because the EPOM is a position that minimizes the depth dependence of the *p*_Q_, the water equivalence of the entrance window, the influence of the cavity, and the dose contribution from the side and back walls can also be considered.^7^ Dosimetry based on the water‐equivalent thickness of the chamber is also performed, but it considers only the effect of the entrance window. Therefore, the EPOM shifted to the center of the cavity, which is a different position from the water‐equivalent thickness. The EPOM is energy‐dependent because the *p*_Q_ change with depth at each energy.^22^, ^23^ For Roos, Zink and Wulff^22^ reported the optimal shift based on a method similar with this study. The results determined using only the data beyond the maximum depth showed shifts of 0.026 and 0.040 cm at 6 and 21 MeV, respectively. This result was very consistent with our study. For NACP‐02, Wang and Rogers^4^ reported Δz at 6 and 22 MeV, with a difference of approximately 0.02 cm, which is similar to our results. For Advanced Markus, Voigth‐Rhetz et al.^5^ investigated the optimal shift from 6 to 18 MeV and determined a maximum shift of 0.007 cm at 6 MeV and a slight shift decrease with the increasing energy. In contrast, the minimum shift in this study was at 6 MeV, and the shift increased with the energy. Advanced Markus has a considerably low sensitive volume compared with other chamber types, and experimental factors such as $k_{S}$ and $k_{pol}$ associated with the energy change can affect the results, yielding a different trend from the MC simulation results. The energy changes reported by Lacroix et al.,^13^ who experimentally determined the EPOM of Roos and NACP‐02, were slightly larger than those in this study and previous studies based on MC simulations. However, it is unclear whether their determined EPOM corrected for depth variations in $k_{S}$ and $k_{pol}$ for the chamber. Further, the plastic scintillation detector has a thicker sensitive volume (1 mm) than the microDiamond detector (1 μm). Therefore, there may be slight difference in the reference data between our study and theirs. The EPOM was determined experimentally in this study, and the energy change was similar to that in previous studies based on MC simulations.

**FIGURE 4:**
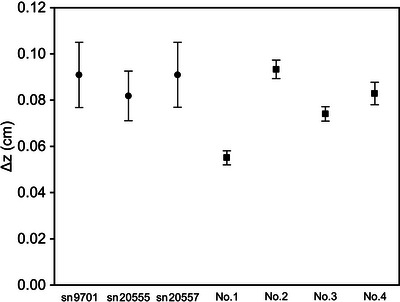
$\Delta z_{\mathrm{meas}}$ (circles) and $\Delta z_{\mathrm{MC}}$ (square) results at 6 MeV. For comparison, in all simulations, Δz was determined with respect to the point 0.06 cm cavity side from the chamber surface. The details of each model are shown in Table 2. The error bars show the uncertainties based on Kawrakow et al.^25^

Even in plane‐parallel chambers with large manufacturing tolerances,^12^ the chamber‐to‐chamber variations in the EPOM were minimal. All serial numbers in Roos agree with the results of previous studies^3^, ^5^, ^13^, ^22^ within the uncertainty, and there is no issue with using the previously reported values. Advanced Markus was slightly larger than in the previous studies,^5^, ^7^ but the variation was small enough that a single Δz could be used for all serial numbers. The difference from previous studies is approximately 0.01 cm, and the Δz determined via MC simulation can be used which can eliminate the influence of experiments, even though additional data are required to determine the optimal shifts in Advanced Markus. We can also use a single Δz for all serial numbers for NACP‐02. However, the issue is what value to use for optimal shifts because it is approximately 0.04 cm larger than that reported by previous studies.^4^, ^5^, ^13^ Chin et al.^24^ reported that NACP‐02 has an uncertainty in the entrance window, and previous studies^4^, ^5^ based on MC simulations have reported the EPOM of the model reproduced as specified. This could be the reason that these results differ from the results of our study. Therefore, the chamber model listed in Table 2 was reproduced by egs_chamber and compared the MC‐determined ($\Delta z_{\mathrm{MC}}$) with the experimental data ($\Delta z_{\mathrm{meas}}$). Model Nos. 2 and 3 are virtual chambers with different entrance window thicknesses and densities from the specifications, reproduced by Wang et al.^4^ and Muir et al.,^7^ respectively, which were also validated in this study. Model No. 4 was reproduced based on the report of Chin et al.,^24^ who actually destroyed the chamber and reported the entrance window details. Consequently, model No. 1 created according to the specifications differed the most from the $\Delta z_{\mathrm{meas}}$. Figure 4 shows $\Delta z_{\mathrm{meas}}$ and $\Delta z_{\mathrm{MC}}$; only 6 MeV was targeted because the optimal shifts depend on the energy. Note that the energy in MC calculation was adjusted so that the beam quality could match that of *R*_50_ measured using the microDiamond detector. Model No. 4 based on Chin's report^24^ was consistent with $\Delta z_{\mathrm{meas}}$. Thus, the NACP‐02 used in this study may have an entrance window thickness, which is different from the specification for all serial numbers. The details of the actual entrance window used in the chamber cannot be confirmed without destruction, as reported by Lacroix et al.^13^ The Δz of NACP‐02 is incorrect at approximately 0.05−0.06 cm from the inner surface of the entrance window, as reported by previous studies; thus, a further shift would be required.

**TABLE 2:** Models of NACP‐02 reproduced by egs_chamber.

Model	Reference	Entrance window (cm)	Graphite layer (cm)	Graphite density (g/cm^3^)	Mylar layer (cm)
No.1	specification	0.060	0.050	1.75	0.010
No.2	Wang^4^	0.090	0.075	1.75	0.015
No.3	Muir^7^	0.060	0.050	2.25	0.010
No.4	Chin^24^	0.067	0.050	2.25	0.017

Optimal EPOM shifts have the potential to reduce the uncertainty in clinical electron dosimetry. Due to the different chamber positioning recommendations given in the national and international dosimetry protocols, the additional uncertainty to the beam quality conversion factor *k*_Q_ ranges from 0.2 to 0.6%.^26^ Note that this is the result of a shift position that considers the entrance window to be water equivalent or electron density equivalent, and not the EPOM that considers the effects of the entrance windows and cavity, as shown in this study. This variation is simply given by the variation of the PDD around the reference depth. Our result shows that at the reference depth, the maximum dose difference between the case of with and without consideration of the optimal shift is 0.3% (6 MeV, NACP‐02). Moreover, Muir et al.^6^ also reported that the optimal EPOM shifts improve the curve fitting accuracy of the *k*_Q_ as a function of *R*_50_ and that the EPOM is expected to be used for absolute dose calibration. The EPOM is also required for relative dosimetry. The conversion from the percentage depth ionization to PDD is only based on stopping power ratios, assuming that the *p*_Q_ are constant with the depth. The optimal EPOM shifts minimize the depth‐dependence variation of *p*_Q_ (closer to constant) in electron beams, which may contribute to the reduction of the uncertainty in the conversion.

This study has two limitations. First, different factors affect the results of the experimental determination of Δz. The accelerator output is stable and there is no setup uncertainty in MC simulation. However, when determining Δz experimentally, the aforementioned factors add to the uncertainty. Additionally, $k_{S}$ and $k_{pol}$ are required to determine the PDD curves of the chamber, and depth‐dependent trends influence Δz. The uncertainties in this study were within 0.015 cm for all the results, and it is difficult to eliminate these factors and reduce uncertainties in experimental determination. This could explain the slight difference between the previous studies based on MC simulations and this study. Second, the number of chamber samples was small. By increasing the number of samples, more details about the chamber‐to‐chamber variations in the EPOM can be clarified. However, even with the three serial numbers used in this study, the variation was still small. Until now, the EPOM has been determined primarily through MC simulation, but there is a possibility that it has an incidence window that differs from the specifications such as NACP‐02. Therefore, the EPOM should be determined experimentally rather through MC simulation.

## 5 CONCLUSION

In this study, the EPOM of the chamber in electron beam dosimetry was experimentally determined. Most of the EPOMs are located inside the cavity and exhibited energy dependence. The chamber‐to‐chamber variations in the EPOMs are small for the three chamber types, enabling the use of a single value. For the Δz of NACP‐02, the use of previously reported values is not recommended. This is because of the uncertainty of the entrance window of NACP‐02. It is necessary to carefully consider where the optimal EPOM is located when using this chamber.

## AUTHOR CONTRIBUTION

All authors have made substantial contributions to either the conception and design of the study, the collection and assembly of the data, or the interpretation of the results. All authors have contributed to the writing of the paper, reviewed successive versions, participated in the revision process, and approved the final version.

## CONFLICT OF INTEREST STATEMENT

The authors declare no conflicts of interest.

## Supporting information

Supporting Information

## Data Availability

The data that supports the findings of this study are available in the supplementary material of this article.
