# Supplementary material for: Experimental investigation of the effective point of measurement for plane‐parallel chambers used in electron beam dosimetry
Source: J Appl Clin Med Phys. 2023 Jun 12;24(7):e14059. doi: 10.1002/acm2.14059 (PMC10338742; doi:10.1002/acm2.14059)
Supplement: Supplementary file 1 — Supporting Information [file ACM2-24-e14059-s001.docx]

Table S1 Summary of the optimal chamber shifts (cm). The numbers in the brackets represent the standard deviation in the last digit. The bold emphases indicate the mean value.

| Chamber | s/n | Optimal chamber shifts (cm) | | | | | | | |
| --- | --- | --- | --- | --- | --- | --- | --- | --- | --- |
|  |  | *R*_50_ = 2.40 | | *R*_50_ = 5.04 | | *R*_50_ = 7.59 | | *R*_50_ = 8.82 | |
| NACP-02 | 9701 | 0.091 | **0.088(5)** | 0.107 | **0.104(5)** | 0.118 | **0.114(6)** | 0.117 | **0.109(8)** |
|  | 20555 | 0.082 |  | 0.098 |  | 0.107 |  | 0.110 |  |
|  | 20557 | 0.091 |  | 0.107 |  | 0.116 |  | 0.100 |  |
| Roos | 994 | 0.020 | **0.026(12)** | 0.035 | **0.043(9)** | 0.037 | **0.046(12)** | 0.047 | **0.046(8)** |
|  | 2915 | 0.040 |  | 0.053 |  | 0.059 |  | 0.053 |  |
|  | 2956 | 0.020 |  | 0.040 |  | 0.041 |  | 0.037 |  |
| Advanced Markus | 413 | −0.004 | **0.001(5)** | 0.011 | **0.015(3)** | 0.022 | **0.022(2)** | 0.007 | **0.009(10)** |
|  | 1878 | 0.002 |  | 0.016 |  | 0.020 |  | 0.000 |  |
|  | 1909 | 0.005 |  | 0.017 |  | 0.024 |  | 0.020 |  |
